# Complete Heart Block Secondary to Microscopic Polyangiitis: A Rare Case Presentation

**DOI:** 10.7759/cureus.8227

**Published:** 2020-05-21

**Authors:** Giuseppe Filice, Ivan Richard, Palak Patel, Jeffery Miskoff

**Affiliations:** 1 Internal Medicine, Hackensack Meridian Ocean Medical Center, Brick, USA; 2 Internal Medicine, Hackensack Meridian School of Medicine at Seton Hall University, Nutley, USA

**Keywords:** complete heart block, microscopic polyangitis, small vessel vasculitis

## Abstract

The potential etiologies of third-degree complete heart block include idiopathic, pathologic, and iatrogenic causes. We present a rare case of complete heart block in an elderly woman with microscopic polyangiitis (MPA), who presented with dyspnea on exertion and dizziness.

## Introduction

Microscopic polyangiitis (MPA), is a small vessel vasculitis, defined as a necrotizing vasculitis, with few or no immune deposits, predominantly affecting small vessels. MPA may be characterized as a pulmonary-renal syndrome with rapidly progressive glomerulonephritis and alveolar hemorrhage; however, the manifestation of the disease varies depending on the organ system involved. The incidence of vasculitis has been on the rise recently. The increasing incidence of vasculitis may be attributed to the development of testing for anti-neutrophil cytoplasmic antibodies (ANCAs), which are found in approximately 90% of all cases of vasculitis [1]. In this study we describe a case of MPA, and review the relevant literature to raise awareness of the prevalence of cardiac manifestations, both clinical and those found on imaging, in the spectrum of ANCA-associated vasculitis. 

## Case presentation

**Figure 1:**
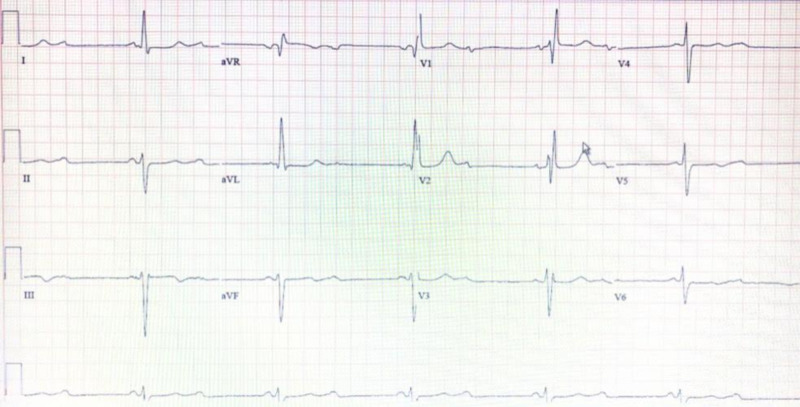
ECG depicting a sinus rhythm at 30 bpm with new onset complete heart block, right bundle branch block, left ventricular hypertrophy, left anterior fascicular block, and complete heart block. ECG, electrocardiogram

A 77-year-old Caucasian female presented to the ER on 19th August, 2019 with dyspnea on exertion and dizziness of two days duration. She had a past medical history of hypertension, hyperlipidemia, diabetes mellitus type 2, coronary artery disease status post 2-vessel coronary artery bypass graft (CABG) in October 2018, hypothyroidism, chronic kidney disease stage 3B with underlying MPA vasculitis on steroids prednisone 2.5 PO daily, colon cancer in 2015 status postchemotherapy, radiation therapy, and lower anterior resection (LAR) with ileostomy and subsequent reversal. The patient also had a history of smoking which she quit 50 years ago. In July 2019, the patient was seen by her primary care physician (PCP) with a chief complaint of bilateral lower extremity pain. Bilateral venous Doppler ultrasound of her lower extremities and a two-dimensional echocardiogram were performed and resulted negative for any acute pathology. On 14th August, 2019 she was found to have a heart rate of 37 bpm. A Holter monitor was placed and her baseline carvedilol was stopped. During this admission, on general examination the patient was awake, alert, oriented, and in no acute distress. Her vitals were as follows: blood pressure (BP) 260/70 mmHg, heart rate (HR) 36 bpm, respiratory rate (RR) 18 rpm, and temperature was 98.7°F. Her breath sounds were clear to auscultation bilaterally. The patient had bradycardia and a systolic murmur 3/6 was appreciated best at the second intercostal space on the right upper sternal border. The patient's abdomen was soft, nontender, and nondistended. There were no focal neurological deficits. Abnormal results of initial laboratory tests were as follows: blood urea nitrogen, BUN 43 (ref. interval 5-25 mg/dL), creatinine 1.68 (ref. interval 0.44-1.00 mg/dL), BUN:creatinine ratio 26 (ref. interval 10-20 mg/dL), glomerular filtration rate (GFR) 30 (ref. interval > 60 mL/min), red blood cells (RBC) 2.63 (ref. interval 4.10-5.10 M/uL), hemoglobin 8.8 (ref. interval 12-16 g/dL), hematocrit 27.3 (ref. interval 35.0%-48.0%), mean corpuscular volume (MCV) 103.8 (ref. interval 80.0-100.0 fL), mean corpuscular hemoglobin (MCH) 33.5 (ref. interval 25.0-30.0 PG), mean corpuscular hemoglobin concentration (MCHC) 32.2 (ref. interval 31.0-36.0 g/dL), and red cell distribution width (RDW) 13.8 (ref. interval 11.5%-14.5%). The patient’s electrocardiogram showed a sinus rhythm at 36 bpm with new onset complete heart block, right bundle branch block, left ventricular hypertrophy, and left anterior fascicular block (Figure 1). She was admitted to the ICU for telemetry monitoring and anti-hypertensive medications were started. Cardiology was consulted and the patient was evaluated by an electrophysiologist for probable permanent pacemaker (PPM) placement. She subsequently had a dual chamber pacemaker placed the next day. The patient was monitored for the following two days, during which she had no further episodes of bradycardia. On the third day, she was discharged home, and was advised to follow up with her cardiologist.

## Discussion

**Figure 2:**
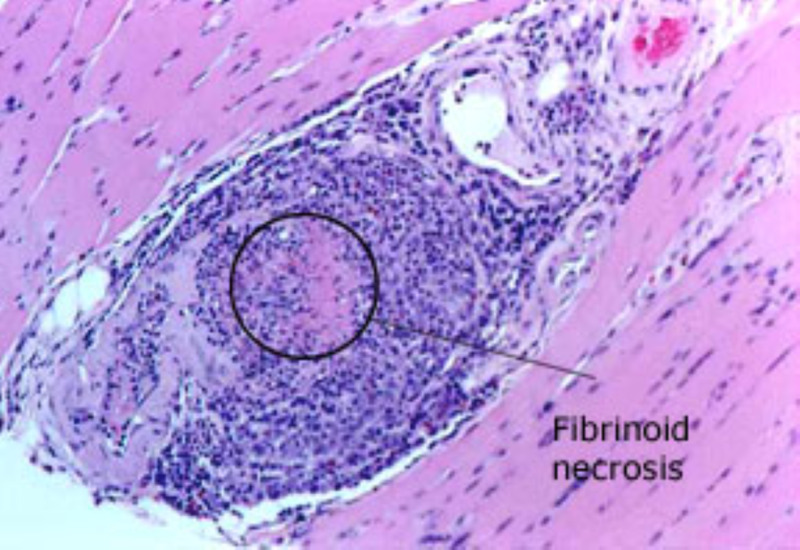
This biopsy of a gastrocnemius muscle in another patient, a 69-year-old man with MPA depicts diagnostic changes of intense inflammatory infiltrate and destruction of the blood vessel wall within the muscle. MPA, microscopic polyangiitis

In this case, our patient was previously diagnosed with MPA and presented with dyspnea on exertion and dizziness. She was diagnosed with a complete heart block found on electrocardiogram (ECG). To our knowledge there are no other cases of MPA-induced complete heart block. Our review of the literature yielded case reports of granulomatosis with polyangiitis (GPA) associated with cardiac conduction abnormalities. This leads us to believe that MPA and GPA exist on a spectrum of small vessel vasculitis instead of two separate diseases. ANCA-associated vasculitis is a group of diseases including MPA and GPA. Diagnosing these conditions poses a significant challenge as the clinical presentation can be similar to malignancy or infection. The common underlying pathology in these diseases is small vessel vasculitis leading to necrosis similar to Figure 2 [2]. Colin et al. presented a case of GPA with endocarditis, pericarditis, myocardial infarction, and atrioventricular block due to infiltration causing a mass to form in the interatrial septum [3]. In GPA, cardiac involvement occurs in up to 44% of patients [4]. Pericarditis and coronary vasculitis are the most frequent findings (50% of cases), but myocarditis, endocarditis, conduction system granulomata, and pulmonary artery involvement have also been reported [3]. Colin et al. supported the use of cardiac MRI to detect cardiac involvement in GPA [3]. Pugnet et al. also supported this notion and in their study of 31 patients with GPA, 61% were found to have some form of cardiac abnormality on cardiac MRI [5]. The most common abnormality the study found was left ventricular (LV) regional wall motion abnormalities (35%) [5]. Arrhythmias (mainly supraventricular), atrioventricular (AV), or bundle branch block can also be found due to myocarditis, ischemia, or heart failure. All the case reports of small vessel vasculitis with cardiac involvement were GPA, which is a rare clinical finding even in that disease state, although 30% were found to have cardiac involvement on autopsy according to Lim-Hing [6]. Bundle branch blocks and heart blocks in GPA can be attributed to granulomatous inflammation of the AV node or bundle branches [6-7].

## Conclusions

In our case of MPA, we attributed the patient's complete heart block to vasculitis-induced necrosis of the coronary arteries given the patient's recent development of coronary artery disease (CAD) requiring coronary artery bypass graft (CABG). We present a rare case of MPA-induced complete heart block. We hope to raise awareness of the prevalence of cardiac manifestations both clinical and those found on imaging, in the spectrum of ANCA-associated vasculitis. 
